# Gold Nanocluster-Promoted Interfacial Electron Transfer of Cytochrome c at an Aqueous–Organic Interface

**DOI:** 10.1021/acs.nanolett.5c05030

**Published:** 2025-12-12

**Authors:** Jose M. Abad, Marcos Pita, Antonio L. De Lacey, Alonso Gamero-Quijano

**Affiliations:** 83076 Instituto de Catálisis y Petroleoquímica (ICP), CSIC, C/Marie Curie 2, 28049 Madrid, Spain

**Keywords:** Gold nanocluster, liquid−liquid electrochemistry, cytochrome c, bioconjugate, bioelectrochemistry

## Abstract

Functional enzyme–gold nanoparticle bioconjugates are becoming increasingly important in bioelectrocatalysis since they facilitate and improve the efficiency of long-range protein interfacial electron transfer between enzymes and electrodes by enhancing conductivity. While much research has focused on solid–liquid interfaces, there is still limited understanding of the key parameters and electrochemical conditions necessary for reliable and reproducible bioelectrochemistry at polarizable aqueous–organic interfaces under native conditions. Herein, we demonstrate how the size of gold-modified nanoparticles influences the interfacial electron transfer of cytochrome c at an aqueous–organic interface. We found that nanoclusters centered in a size of 1.2 nm, equivalent to the water-trifluorotoluene mixed solvent layer (ca. 1.5 nm), work in tandem with cytochrome c to facilitate oxygen reduction reactions. In contrast, bioconjugates comprising larger gold nanoparticles are less effective in enhancing cytochrome c electrochemistry, with the gold nanoparticles acting as independent catalysts at the interface.

## Graphical abstract

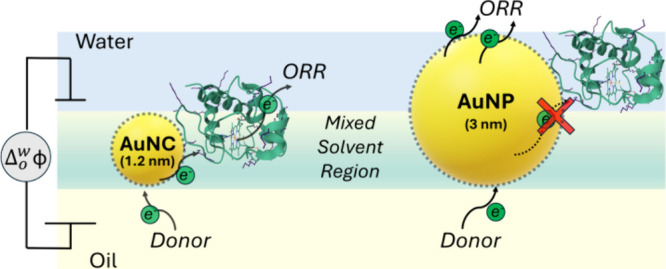

A challenge in bioelectrochemistry and molecular bioelectronics is obtaining efficient interfacial electron transfer (ET) of redox enzymes at electrode surfaces. Enzymes’ ET often depends on their precise orientation, i.e. how close their redox centers are to the electrode surface, and how well preserved their native conformation is upon immobilization. Gold nanoparticles have been employed in different works to bridge the electron transfer gap between enzyme with electrode through enhanced conductivity and molecular recognition,^1–3^ improving the efficiency of long-range protein interfacial ET. Ulstrup et al.^4^ demonstrated that for cytochrome c (Cyt c) this can be achieved by gold nanoparticle (AuNP)-assisted assembly on macroscopic single crystalline electrode surfaces. AuNPs appeared to serve as excellent ET relays, most likely by facilitating the electron hopping between the protein redox center and the electrode surface. While all this research has been carried out at solid–liquid interfaces, to date, no work has been reported at a polarizable liquid–liquid interface, and little is known about the parameters and electrochemical conditions to perform reliable and reproducible interfacial bioelectrochemistry. This biphasic system, where the interface is formed by two immiscible liquid phases—most commonly aqueous and organic electrolyte solutions—constitutes a biomimetic platform that provides a soft, tunable environment for studying ET processes of proteins.^5,6^

Here, we report the size-dependent enhancement of interfacial ET of Cyt promoted by gold clusters at an aqueous–organic interface. Hybrid systems formed by Cyt c coupled to gold clusters of 1.2 nm and gold nanoparticles of 3 nm, were studied. First, monolayer-protected gold nanoclusters capped with Thioctic Acid (TA-AuMPCs) of a size (1.2 nm) comparable to the thickness of an interface formed by phosphate buffer (W) and trifluorotoluene (TFT) (ca. 1.5 nm)^7^ were synthesized ex-situ (Figures S1 and S2). Afterward, they were bioconjugated with Cyt c through electrostatic interactions between the negatively charged surface of the TA-AuMPCs and positively charged regions of Cyt c, such as sites A or L,^8,9^ which contain lysine residues. Further details about the synthesis and characterization are provided in the Supporting Information. The purified Cyt c-TA-AuMPCs were characterized by UV–vis spectroscopy (Figure S5). The Soret band of Cyt c, which results from π–π* electronic transitions in the porphyrin ring of the heme group, was observed at 409 nm and remained unchanged, indicating that no conformational changes in the heme pocket occurred after coupling to gold clusters, consistent with previous reports by Ulstrup et al.^4^ The formation of the Cyt c-TA-AuMPCs bioconjugate, hereafter 1.2 nm-bioconjugate, was further corroborated by electron microscopy using HAADF-STEM (Figure 1).

**Figure 1:**
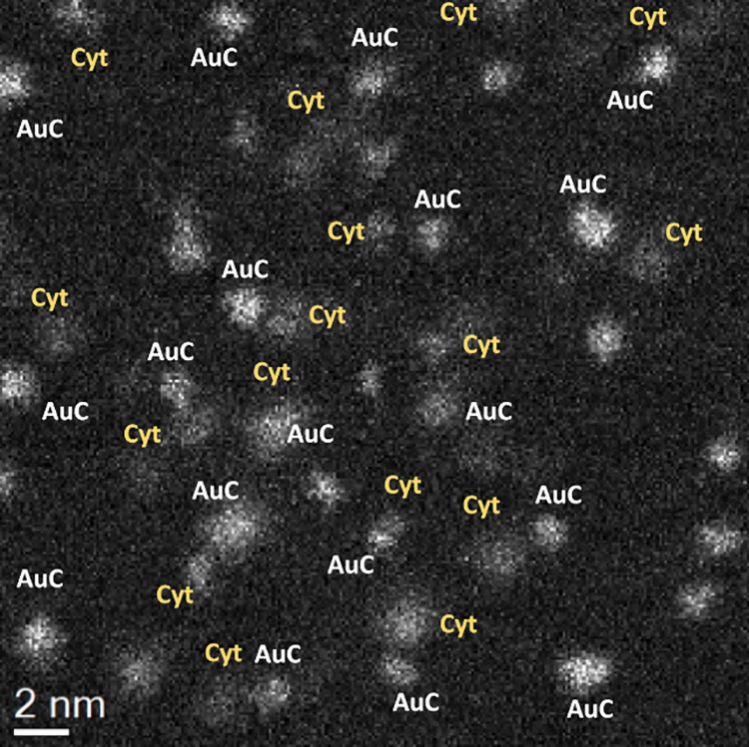
HAADF-STEM of Cyt c-TA-AuMPCs (1.2 nm bioconjugate).

The image obtained after complete removal of unbound Cyt c by purification shows Au clusters paired to Cyt c, where only one cluster per protein was observed. It indicates a defined coupling site, presumably between the carboxyl groups on the AuNP and a preferred binding site in Cyt c (*vide infra*).

The bioconjugates were further characterized electrochemically at a polarizable aqueous–organic interface by AC (differential capacitance) and DC (cyclic voltammetry) methods. First, the assembly and interactions of the bioconjugate with the interface were confirmed through differential capacitance measurements using cell I configuration (Scheme 1).

**Scheme 1:**
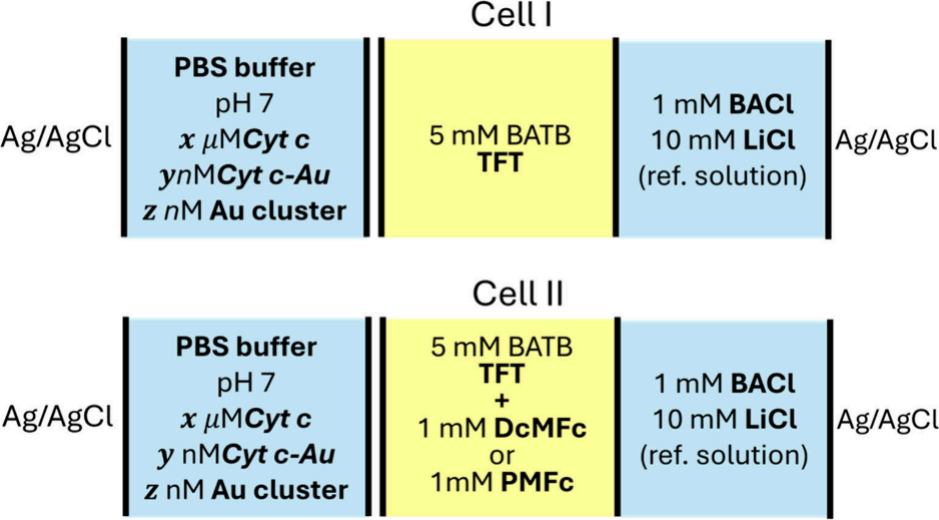
Electrochemical Cell Configurations of the Aqueous (Light Blue)-Organic (Yellow) Interface^a^

a Cell I configuration was used for differential capacitance measurements. Cell II configuration was used to evaluate the electrocatalytic performance of Cyt c-TA-AuMPCs clusters of 1.2 nm and AuNPs of 3 nm. All the electrochemical experiments were performed using a 4-electrode electrochemical cell. Further details about the 4-electrode cell setup are given in the SI.

The differential capacitance curves provide a snapshot of the interfacial charge distribution at a given potential in the presence of charged or zwitterionic species.^10^ A minimum change in the capacitance profile or shifting of the potential of zero charge (PZC) indicates adsorption of the species with a defined orientation and charge. In the presence of the Au clusters, the positive shift of the PZC (ca. + 0.035 V) indicated that the clusters present an apparent negative net charge (Figure S6A, black curve) due to the deprotonation of the carboxylic group of thioctic acid at pH 7. For either Cyt c or the bioconjugate, the PZC shifted toward negative potentials (ca. −0.088 V), indicating an apparent positive net charge (Figure S6). The net charge of Cyt c has been reported as +8 at pH 7,^11^ which is consistent with a negative shift of the PZC observed. On the other hand, the apparent positive net charge of the bioconjugate suggested that some positive regions of the protein remained in contact with the interface. The optimal bioconjugate concentration in the aqueous phase was estimated to be 104 nM, based on the maximum shift of the PZC measured and its stabilization above this concentration (Figure S7). This suggested limited free space for additional bioconjugates as their concentration increased in the aqueous phase. A higher concentration will result in the saturation of the interface,^12^ leading to the loss of interfacial plasticity and the formation of interfacial multilayers of bioconjugates, which could potentially hinder the study of interfacial electron transfer reactions at polarizable aqueous–organic interfaces (*vide infra*).

The 1.2 nm gold clusters and the bioconjugates’ electrocatalytic activity for Oxygen Reduction Reactions (ORRs) were evaluated by cyclic voltammetry measurements using the electrochemical cell II configuration (see Scheme 1). In this setup, 1 mM decamethylferrocene (DcMFc^+^/DcMFc, E°: 0.107 V vs. SHE)^10^ serves as the electron donor and is dissolved in the organic phase. The 1.2 nm gold clusters alone (Cyt c-free) did not generate electrochemical current signals indicative of ORR activity at a polarizable aqueous–organic interface, as evidenced by the cyclic voltammograms in Figure S8. In contrast, the highest ORR currents were recorded with the bioconjugate, indicating a synergistic interaction between the protein and the gold clusters. The cyclic voltammograms at varying bioconjugate concentrations are shown in Figure 2A. The positive current observed from 0.1 V toward more positive potentials was attributed to the production of ROS, mainly H_2_O_2_ species.^13^ A positive Galvani potential significantly increases the interfacial interactions between Cyt c and the organic anion TB^–^. These interactions result in greater exposure of the heme center to both the Au clusters and the interface, thereby increasing the probabilities of electron transfer between electron donors and the protein. The maximum ORR current was observed with a concentration of bioconjugates of 305 nM (Figure 2A, black curve). The negative peak current observed at negative potentials was ascribed to the DcMFc^+^ mass transfer from the organic to the aqueous phase, which is generated during ROS production. A higher concentration of bioconjugates, e.g., 1.5 μM, was detrimental and decreased the ORR and DcMFc^+^ currents (Figure 2A green curve). Such decay should be attributed to interfacial fouling caused by bioconjugate aggregation at the aqueous–organic interface and the concomitant lower access of O_2_ to the interface. Crucially, the bioconjugates presented higher electrocatalytic activity toward ORR compared to the activity observed with 10 μM Cyt c in bulk solution (see Figure.S9).

**Figure 2:**
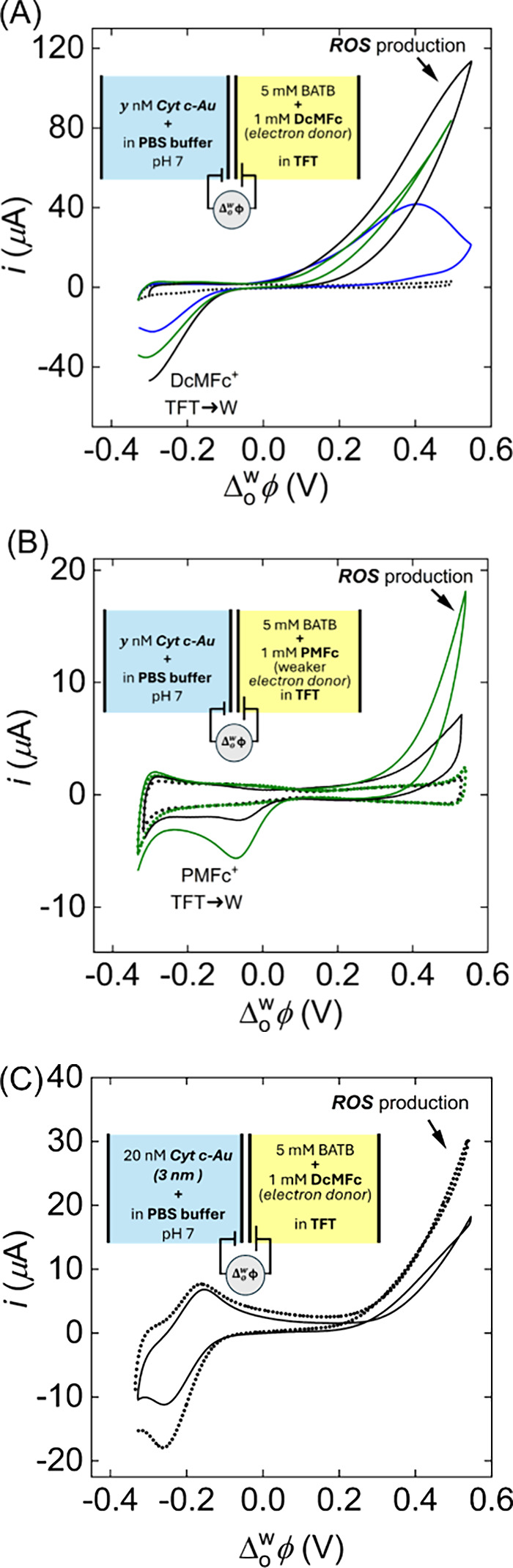
Representative cyclic voltammograms (CVs) of Reactive Oxygen Species (ROS) production at an aqueous–organic interface promoted by the presence of 1.2 nm- and 3 nm-bioconjugates with DcMFc or PMFc as electron donors. (A) CVs varying the concentrations of the 1.2 nm-bioconjugates, (*y*) nM: 0 (dotted line), 109 nM (blue line), 305 nM (black line), and 1.5 μM (green line). (B) ROS production using a weak electron donor such as pentamethylferrocene (PMFc). The dotted lines are blank voltammograms before adding the 1.2 nm -bioconjugates (green dotted curve) and Cyt c (black dotted curve). The ROS production in the presence of 204 nM 1.2 nm-bioconjugates (green curve) was 3-fold higher than the currents recorded with 10 μM Cyt c (black curve). (C) CVs in the presence of 1.6 nM 3 nm-TA-AuNps (dotted line) and 20 nM 3 nm-bioconjugates (solid line). Scan rate: 20 mV s^–1^.

The electrocatalytic performance of the 1.2 nm-bioconjugates was confirmed using a weaker electron donor such as pentamethylferrocene (PMFc^+^/PMFc, E°: 0.415 V vs. SHE)^10^ in the organic phase (cell II configuration,Scheme 1). The electron transfer from PMFc to Cyt c was observed despite the reduction potential of Cyt c (E°: 0.246 V vs SHE)^14^ being lower than that of PMFc. While the applied Galvani potential does not provide a driving force to facilitate the thermodynamically uphill reactions it alters the local environment of the interface (e.g., dielectric constant, ionic strength, and local pH).^13,15^ Here, the standard reduction potential of the weaker donor decreases when larger positive Galvani potentials are applied, however not only is the electron donor affected by the potential, but also the metallic surface of the cluster, which acts as a bipolar-like electrode. As a result, the reaction between the electron donor (DcMFc or PMFc) and the protein will consistently be enhanced by the presence of the Au cluster, as demonstrated by the larger currents in the CVs. Indeed, the ORR current with the 1.2 nm-bioconjugates was 3-fold higher than that measured with Cyt c in solution (Figure 2B). Such larger ORR currents were obtained with a nanomolar concentration of Cyt c (10-fold lower) compared to a micromolar concentration in solution.

In order to evaluate the particle size dependence on the ORR currents, 3 nm-TA-AuNPs were prepared (Figures S3 and S4). Their addition into the aqueous phase did not produce changes in the capacitive profiles, and no shift of the PZC was observed (see Figure S10). Crucially, the 3 nm-TA-AuNPs showed high electrocatalytic activity toward ORR (see Figure S11) compared to the negligible activity seen with 1.2 nm-TA-Au-clusters (Figure S8) at neutral pH. This finding suggests that only a few-nanometer-sized NPs (3 nm ≤ ) should be used to prepare bionconjugates in order to avoid large background ORR currents. The electrocatalytic inactivity of 1.2 nm-TA-Au-clusters may be attributed to their discrete electronic structure, in contrast to larger Au nanoparticles, which have a continuous electronic structure that favors their electrocatalytic activity for ORR under an external bias.^16^

The 3 nm TA-AuNPs were further bioconjugated with Cyt c (UV–vis spectrum shown in Figure S12). The presence of the 3 nm-bioconjugates at the polarizable aqueous–organic interfaces was detected by differential capacitance measurements by a small shift of the PZC of ca. 0.01 mV, indicating that the bioconjugates presented an apparent positive charge (see Figure S13). The shift of the PZC was less pronounced compared to 1.2 nm-bioconjugates, suggesting that the Cyt c had less contact with the interface. This was confirmed by the 3 nm-bioconjugate’s lack of interfacial activity toward ROS production, suggesting that the heme center was less exposed toward the interface (Figure 2C). The ORR currents measured with 3 nm-bioconjugates were lower than those observed with only the 3 nm TA-AuNPs, as shown in Figure 2C. These results suggest that the electron transfer pathway for ROS production occurs mainly through the 3 nm-AuNPs, and the synergy observed between Cyt c and Au was lost. Schematics of our findings are shown in Figure 3. Few-nanometer-size (≤1.2 nm) bioconjugates (Figure 3A) still permit the contact of the protein with the interface and enable the heme to be exposed through interactions with TB^–^ organic anions at the aqueous–organic interface. On the other hand, bioconjugates with nanoparticles of larger size inhibited Cyt c’s electrocatalytic activity (see Figure 3B). Furthermore, the electrons preferred another pathway to reduce oxygen through the metallic side of the 3 nm-bioconjugates. The size of the 3 nm AuNPs must restrict Cyt c’s interaction with TB^–^ species, which is a crucial factor in partially exposing the heme center and activating its enzymatic function.

**Figure 3:**
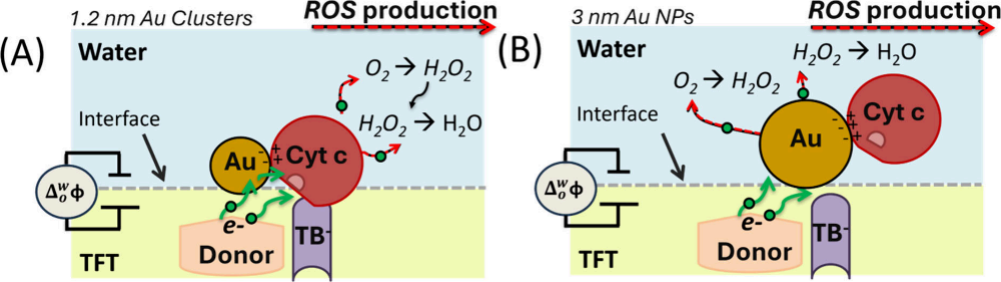
(A,B) Schematic representation of the bioconjugates assembled at a polarizable aqueous–organic interface.

Summarizing, the electrocatalytic activity of Cyt c bioconjugates depends on the size of the clusters, and results in a crucial parameter to consider when synthesizing electroactive bioconjugates to perform experiments at aqueous–organic interfaces. The use of Au clusters with a diameter similar to that of the mixed solvent layer (ca. 1.5 nm) of a water-trifluorotoluene interface permits the interactions between the protein and the interface. These results demonstrate a significant improvement, given that the Cyt c concentration in the bioconjugates was 32-fold lower than the state-of-the-art concentration of 10 μM in the aqueous bulk phase.^17^ It is worth noting that most reports have been conducted with Cyt c concentrations in the micromolar range. Therefore, the use of bioconjugates allows spending less amount of protein. Bioconjugates with AuNPs of larger size than 3 nm inhibit the direct electron transfer between donor and Cyt c at the aqueous–organic interface.

Our findings open up opportunities to explore novel electron donors with lower driving forces beyond those traditionally used at aqueous–organic interfaces, such as methylated ferrocenes. The use of a few-nanometer-sized clusters in the bioconjugates could enable the feasibility of conducting thermodynamically uphill reactions at a polarizable aqueous–organic interface, where the Au-cluster could act as a bipolar-like electrode decreasing the overpotential of the electron transfer. On the other hand, the electrocatalytic activity of a few-nanometer-sized (3 nm ≤ ) AuNPs capped with thioctic acid (Cyt c-free) are promising catalysts toward ORR at a polarizable aqueous–organic interface under physiological conditions, as shown by the larger currents achieved at higher potentials.

## Supplementary Material



## Notes

Experimental details, materials, and methods, including STEM images and UV–vis spectra of 1.2 nm TA-AuMPCs and 3 nm TA-AuNPs, UV–vis spectra of bioconjugates Cyt c-TA-AuMPCs and Cyt c-TA-AuNPs, differential capacitance curves of a polarizable aqueous–organic interface before and after adsorption of Cyt c-TA-AuMPCs and Cyt c-TA-AuNPs bioconjugates, TA-AuMPCs, TA-AuNPs, Cyt c at different concentrations, and cyclic voltammograms of TA-AuMPCs, TA-AuNPs, Cyt c and the Cyt c-TA-AuNPs bioconjugate at an aqueous–organic interface (PDF)
