# Supplementary material for: Gold Nanocluster-Promoted Interfacial Electron Transfer of Cytochrome c at an Aqueous–Organic Interface
Source: Nano Lett. 2025 Dec 12;25(51):17814–8. doi: 10.1021/acs.nanolett.5c05030 (PMC12751104; doi:10.1021/acs.nanolett.5c05030)
Supplement: Supplementary file 1 [file nl5c05030_si_001.pdf]

Supporting Information

Gold Nanocluster-Promoted Interfacial Electron Transfer of Cytochrome C at Aqueous-Organic Interface

Jose M. Abad,* Marcos Pita, Antonio L. De Lacey and Alonso Gamero-Quijano*

Instituto de Catálisis y Petroleoquímica, CSIC. C/Marie Curie 2, 28049 Madrid, Spain.

Experimental section

1. Chemicals

All chemicals were used as received without further purification. The organic solvent α,α,α-trifluorotoluene (TFT, 99%+), was received from Fluorochem. The TFT solvent was stored in a flask with activated molecular sieves with a pore size of 3Å obtained from Honeywell FlukaTM. The purity of the TFT solvent was tested before use by cyclic voltammetry (CV) and differential capacitance measurements at a polarizable aqueous-TFT interface. Bis(triphenylphosphoranylidene) ammonium chloride (BACl, 97%), lithium chloride (LiCl, ≥99%), Hydrogen tetrachloroaurate(III) trihydrate ($HAuCl_4 \cdot 3H_2O$, 99.99%), Sodium borohydride ($NaBH_4$, 99.99%), Sodium citrate trisodium salt dihydrate, Tetraoctylammonium bromide (TOABr), Thioctic acid (TA, 1,2-dithiolane-3-pentanoic acid), N-(2-Hydroxyethyl)piperazine-N′-(2-ethanesulfonic acid) (HEPES, ≥99.5% BioUltra), Decamethylferrocene (DcMFc, 97%) were purchased from Sigma-Aldrich. Pentamethylferrocene (PMFc) was prepared as reported elsewhere.[1] Lithium tetrakis(pentafluorophenyl)borate diethyletherate (LiTB) was received from Boulder Scientific Company (99%). The organic electrolyte salt of bis(triphenylphosphoranylidene)ammonium tetrakis(pentafluorophenyl)borate (BATB) was prepared by metathesis of equimolar solutions of BACl and LiTB in an ethanol-water (2:1, v/v) mixture. The resulting precipitates were filtered, washed, and recrystallized from acetone. The BATB crystals were recovered and soaked in a mixture of acetone-water (1:1, v/v) to remove all remaining impurities. The purified BATB crystals were filtered, dried in the oven at 37ºC, and stored in a silica-gel desiccator. pH 7 phosphate buffer solutions were prepared with potassium dihydrogen phosphate and sodium hydrogen phosphate purchased from Sigma Aldrich. All aqueous solutions were prepared with ultrapure water (Millipore Milli-Q; specific resistivity, 18.2 MΩ·cm).

2. **Synthesis of the Thioctic Acid Monolayer protected gold clusters (TA-AuMPCs)**

The clusters were prepared by the two-phase method of Brust et al.[2] with the modifications previously reported but using a 3:1 Thioctic acid-to-$HAuCl_4$ molar ratio.[3] While, higher or lower molar ratios yield smaller or bigger gold clusters, the ratio 3:1 was found to be suitable for obtaining a gold cluster of equivalent size to the thickness of the mixed solvent layer (1.2 nm) at our aqueous-organic system.[4] Typically, $AuCl_4^-$ was transferred from an aqueous solution (6 mL, 30 mM, $HAuCl_4 \cdot 3H_2O$) to the organic phase using TOABr in toluene as the phase-transfer reagent (16 mL, 50 mM) with vigorous stirring for 30 min.

Following, TA (2 mL, 270 mM in toluene) was then added to the two-phase mixture and stirred for another 30 min. Finally, a freshly prepared cooled aqueous solution of $NaBH_4$ (5 mL, 0.4 M) was rapidly added to the solution mixture at ~3 °C under vigorous stirring. The organic layer turned cloudy brown, and the mixture was left stirring for 3 h at ~3 °C. The clusters formed after reduction were soluble in the aqueous phase due to their carboxylate termination, and the organic phase was discarded. Scanning transmission electron microscopy (STEM) characterisation of the synthesized TA-AuMPCs (Fig. S1) shows a narrow cluster size distribution with an average diameter of 1.2 ± 0.2 nm. The UV-vis spectrum (Fig. S2) did not show the characteristic plasmon band at 520 nm, confirming particle diameters below 2 nm. A TA-AuMPCs concentration of 4.1 x 10^{16} clusters mL^{-1} was estimated by Total reflection X-ray fluorescence spectrometry (TXRF).

3. **Synthesis of 3nm-Thioctic Acid gold nanoparticles (TA-AuNPs)**.

3 nm-TA capped-gold nanoparticles were obtained by a two-step approach. In the first step, citrate stabilized-gold nanoparticles (Cit-AuNPs) were prepared by reducing $HAuCl_4$ by $NaBH_4$ at the presence of trisodium citrate in water, as follows: In a 25 mL round bottom flask in ice, 238 microliters of 25 mM $HAuCl_4$ were added to 12 mL of H_2O under stirring. Following, 265 μL of 60 mM sodium citrate aqueous solution were injected. One minute later, 79.5 μL of a freshly prepared and cooled 0.1 M aqueous $NaBH_4$ solution, was rapidly added to the solution mixture at ~3 °C under vigorous stirring. A brown-red coloration in solution appeared, indicating the formation of colloidal gold nanoparticles. The colloidal solution was stirred additionally for 30 min.

The second step was the replacement of the synthesized Cit-AuNPs by adding TA into the aqueous solution of colloidal gold. Typically, 20 μL of 10 mM of a TA solution prepared in EtOH were added to 2.0 mL of Cit-AuNPs preadjusted to pH 11 by NaOH. The gold solution in a 2 mL Eppendorf tube was stirred overnight (12-18 h) at room temperature. The TA-functionalized nanoparticles were then purified by ultrafiltration through low-adsorption hydrophilic 30000 NMWL cut-off membranes (regenerated cellulose, Amicon) to remove replaced citrate and excess of TA. Six cycles of centrifugation were carried out at 8000 rpm for 8 min at 15ºC employing 20 mM HEPES buffer (pH 7.5). UV-Vis characterization (Fig. S3, TA-AuNPs diluted 10.5-fold) showed the characteristic localized surface plasmon resonance (LSPR) band at 510 nm, which corresponded to a particle diameter of 2.9 nm estimated using the equation reported by Haiss et al.[5] This size was verified using STEM microscopy (Fig. S4), which measured a value of 2.9 ± 0.8 nm. A molar and particle concentration of 1.8 μM and 1.1×10^{15} particles/mL, respectively were determined from LSPR (Fig. S3), using the Lambert-Beer equation and an extinction coefficient: $\varepsilon_{SPR}=1.76\ x10^{6}\ M^{-1}\cdot cm^{-1}$.[6]

4. Preparation of Cyt c/AuMPs and Cyt c/AuNPs bioconjugates.

Once the TA-AuMPCs and TA-AuNPs were synthetized, they were incubated with a 30x and 5x excess of Cyt c in 25 mM HEPES buffer pH 7.5 overnight at 4ºC, respectively. The bioconjugates were purified by ultrafiltration through low-adsorption hydrophilic 30000 NMWL cut-off membranes (regenerated cellulose, Amicon) to remove excess of Cyt c.

5. Transmission electron microscopy.

Scanning transmission electron microscopy (STEM) imaging was carried out in a probe-corrected Titan (Thermofisher, formerly FEI) operated at 300 kV and equipped with a high brightness X-FEG and a spherical aberration Cs-corrector (CEOS) for the condenser system to provide sub-angstrom probe size. High angle annular dark field (HAADF) images were obtained with a HAADF detector by Fischione.

6. Total reflection X-ray fluorescence spectrometry (TXRF).

TXRF analysis of the samples was performed with a benchtop S2 PicoFox TXRF spectrometer from Bruker Nano GmbH (Germany), equipped with a molybdenum X-ray

source working at 50 kV and 600 μA, a multilayer monochromator with 80% reflectivity at 17.5 keV (Mo Kα), an XFlash SDD detector with an effective area of 30 mm2, and an energy resolution better than 150 eV for Mn Kα.

7. Electrochemical measurements

All the glassware was cleaned by soaking in a permanganate acidic solution of 0.2M $KMnO_4$ + 0.2M H_2SO_4 for at least 4 hours, followed by rinsing with diluted Piranha solution (0.2 M Sulfuric acid and 60 mM H_2O_2) and ultrapure water, 0.055 $\mu S \cdot cm^{-1}$. The final step was a rinse with *aqua regia* (3:1 molar ratio of hydrochloric acid to nitric acid), to remove any adsorbed gold clusters on the glass walls and a final rinse with ultrapure water (Caution! use extreme care when using these corrosive solutions!). Then the electrochemical cells were dried in an oven overnight. The geometrical area of the aqueous-organic interface was estimated as 1.53 cm^2. Differential capacitance and cyclic voltammetry measurements were performed with a Wavedriver 200 bipotentiostat (PINE Research Instrumentation, USA). Data was recorded with Aftermath Software ver.1.6.1. Differential capacitance measurements were performed at a frequency of 80 Hz and a Root Mean Square amplitude (RMS) of 10 mV. Cyclic voltammetry measurements were performed at a scan rate of 20 mV s^{-1}. The scan direction for both techniques was from negative towards positive potentials.

8. 4-electrode electrochemical cell

Liquid-liquid interface electrochemical studies require a 4-electrode configuration which use two pseudo-reference Ag/AgCl electrodes. The aqueous pseudo-reference electrode is stable in most of the aqueous solutions tested, and it is placed directly in the aqueous phase. In contrast, the organic pseudo-reference is less stable when it is in contact with the organic solution (BATB precipitation), and thus, in biphasic systems, an aqueous reference electrolyte solution (ref. solution) is usually added to avoid the direct contact of the organic pseudo-reference with the organic phase. This additional organic-aqueous junction is calibrated with a standard ion probe such as tetraethylammonium chloride (TEA^+). The phosphate buffer solution composition was 60 mM sodium hydrogen phosphate + 20mM KH_2PO_4.

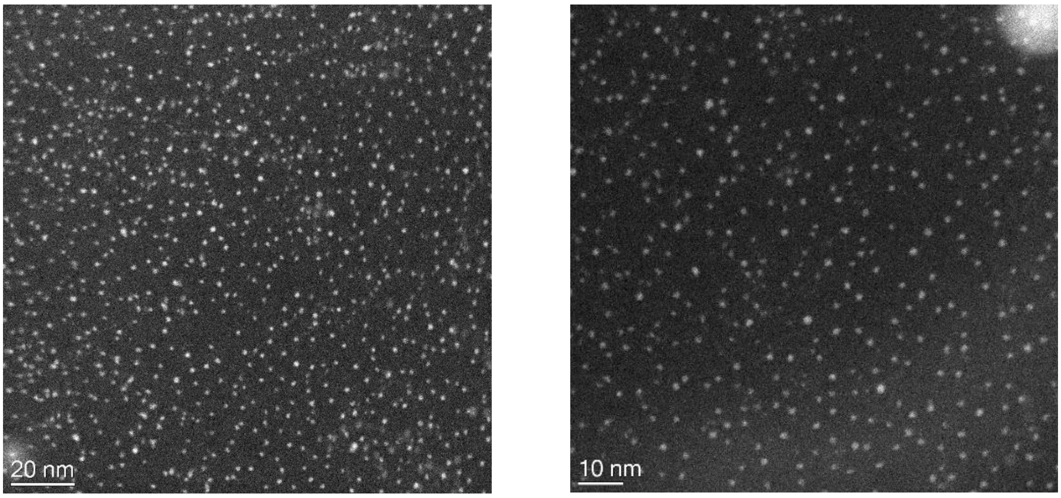

Figure S1. STEM images of 1.2 nm TA- gold clusters in water at different magnifications.

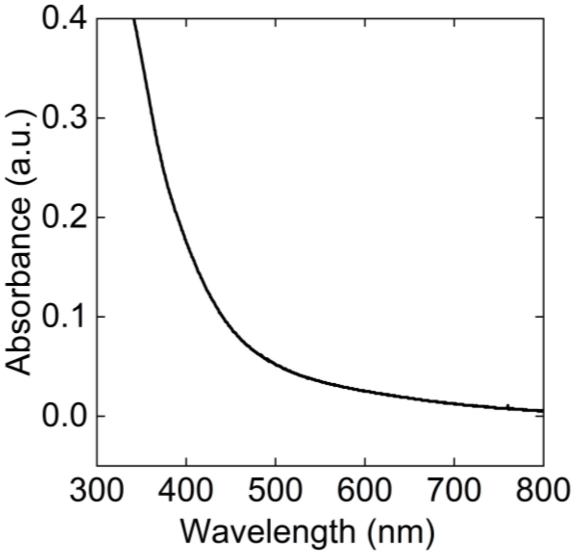

Figure S2. UV-Vis spectrum of 1.2 nm TA-gold clusters in 20 mM HEPES buffer, pH 7.5.

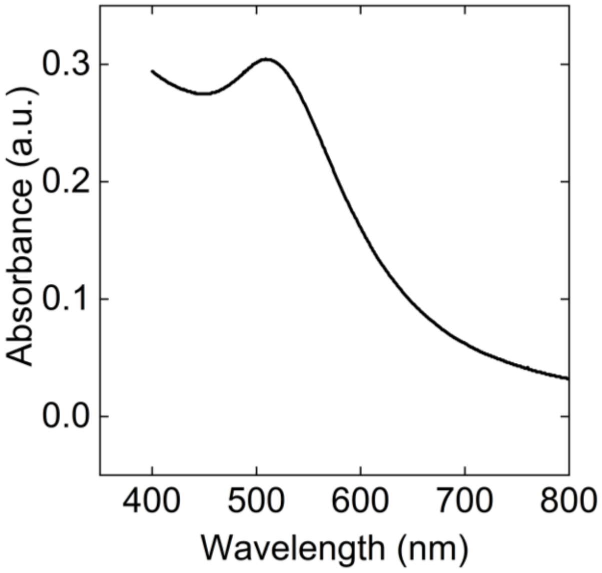

Figure S3. UV-Vis spectrum of 3 nm TA- gold nanoparticles diluted 10.5-fold in 20 mM HEPES buffer, pH 7.5.

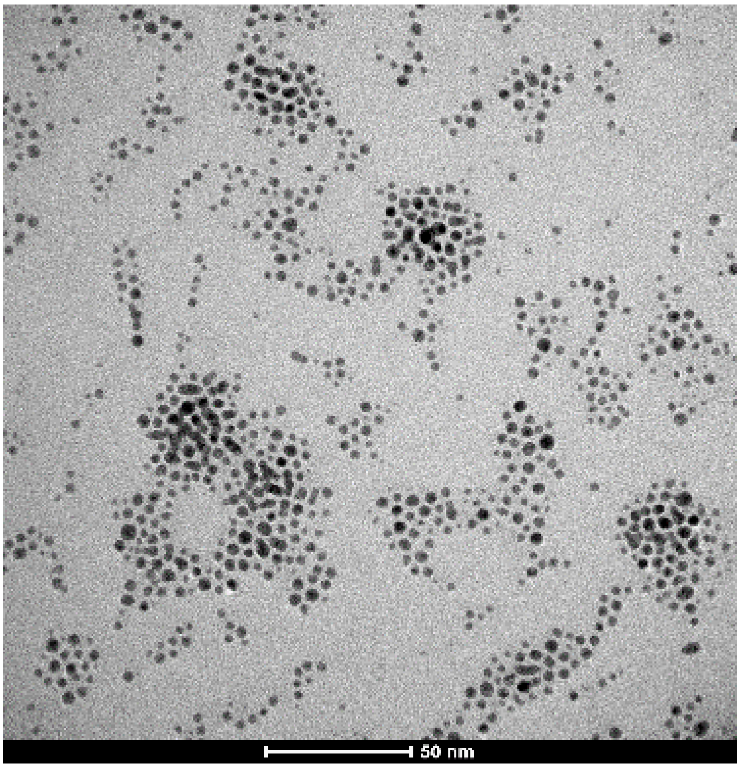

Figure S4. STEM image of 3 nm TA-AuNPs in water.

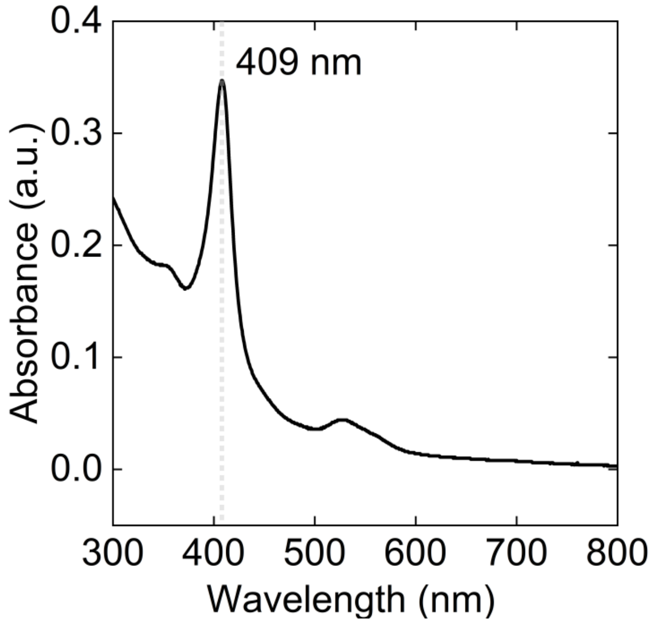

Figure S5. UV-vis spectrum of the bioconjugate Cyt c-TA-AuMPCs (1.2nm) in 40 mM HEPES buffer, pH 7.5. Soret band centered at 409 nm and Q-band between 500 and 560 nm.

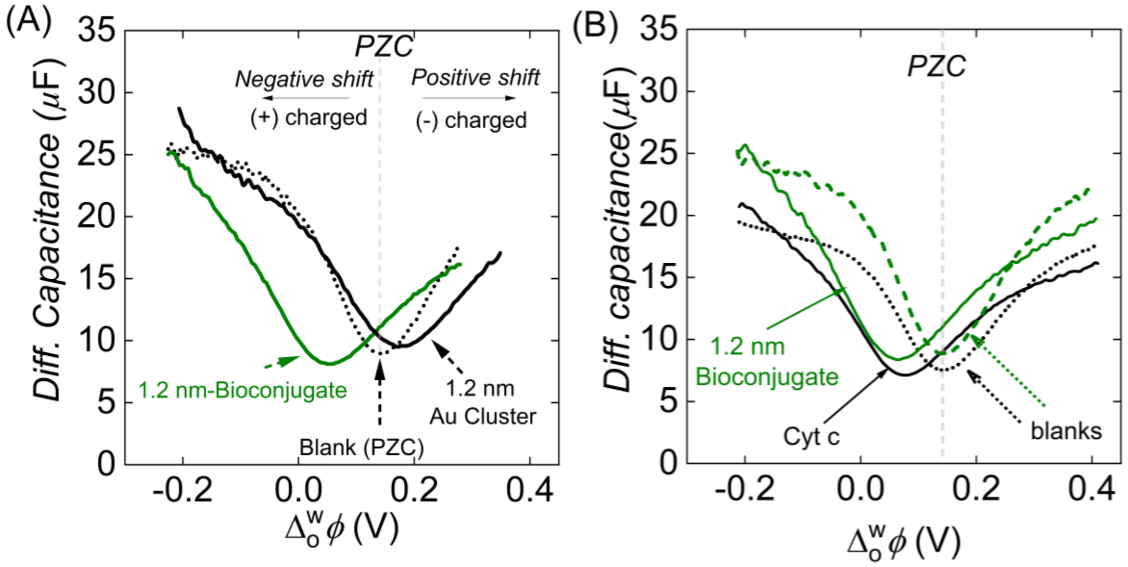

Figure S6. (A) Differential capacitance curves of a polarisable aqueous-organic interface before ($\boldsymbol{x}, \boldsymbol{y}, \boldsymbol{z}$: .0 mM, black dotted curves) and after the adsorption of $\boldsymbol{z}$: 104 nM of 1.2 nm bioconjugates (black curve) and $\boldsymbol{y}$: 226 nM TA-AuMPCs (green curve). (B) Differential capacitance curves of a polarisable aqueous-organic interface before ($\boldsymbol{x}, \boldsymbol{y}, \boldsymbol{z}$: .0 mM, black and green dotted curves) and after the adsorption of $\boldsymbol{z}$: 109 nM 1.2 nm bioconjugates (black curve) and $\boldsymbol{x}$**:** 106 nM Cyt c bulk solution (green curve). These experiments were performed using cell I configuration.

When charged species are present at the interface, they alter the charge distribution, causing the potential of the minimum capacitance (a.k.a., potential of zero charge (PZC))

to shift either positively or negatively, which is related to the apparent net charge of the species present at the interface. The differential capacitive curve of a bare aqueous-organic interface is shown as a dotted curve in Fig. S6A, with a PZC centred at 0.14V. In the presence of the Au clusters, the positive shift of the PZC (ca. + 0.035 V) indicated that the clusters present an apparent negative net charge (see Fig. S6A, black curve) due to the deprotonation of the carboxylic group of thioctic acid at pH 7. Additionally, this shift aligns with the negative zeta potential of the TA-AuMPCs, which has been reported to range from -35 mV to -50 mV by Haes *et al.*[7]

The presence of gold clusters slightly increased the differential capacitance profile compared to the drop observed with Cyt c in solution or the bioconjugate (see Fig. S6 A and B).

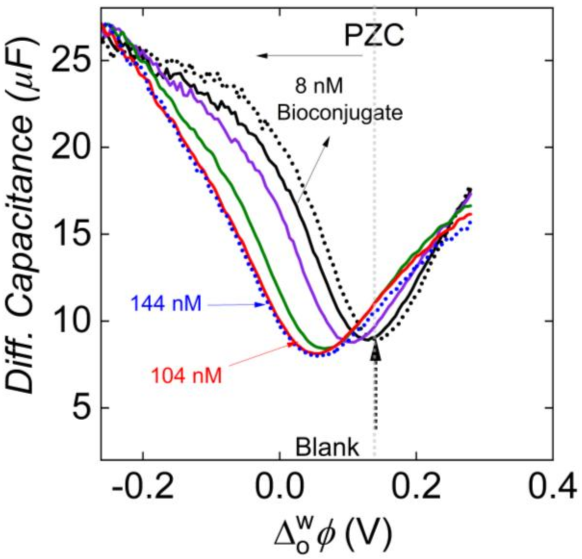

Figure S7. Differential capacitance curves of a polarisable aqueous-organic interface before ($\boldsymbol{x, y, z}$:.0 mM, black dotted curve) and after the adsorption of different concentrations of Cyt c-TA-AuMPCs 1.2 nm bioconjugates: 8 nM (black curve), 24 nM (purple curve), 64 nM (green curve), 104 nM (red curve) and 144 nM (blue doted curve)

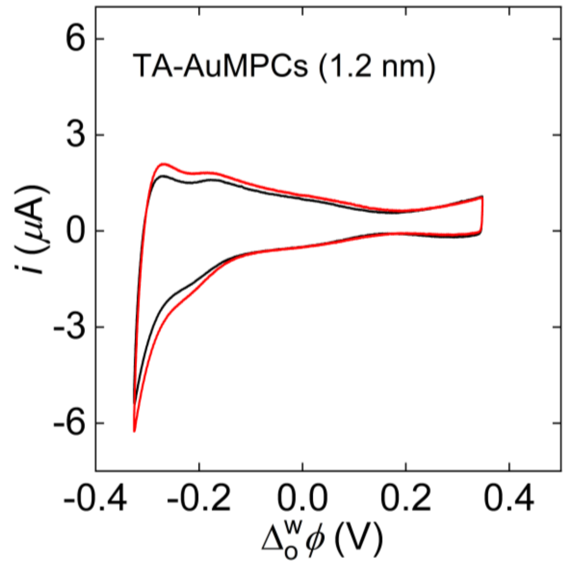

Figure S8. Cyclic voltammograms of 1.2 nm gold clusters (Au Cyt c-free) at an aqueous-organic interface and DcMFc in the organic phase as electron donor. Scan rate 20 mV s^{-1}. Electrochemical cell 2 configuration, *z*: 68 nM (black curve) and 226 nM (red curve).

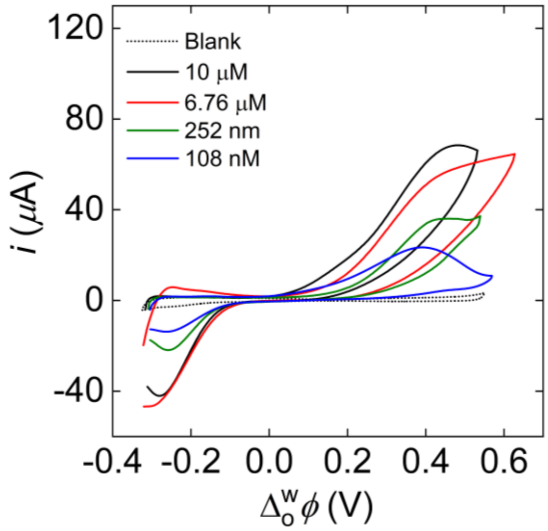

Figure S9. Representative cyclic voltammograms of an aqueous-organic interface in the presence of aqueous Cyt c at different concentrations and 1mM DcMFc in the organic phase as electron donor. Scan rate: 20 mV s^{-1}.

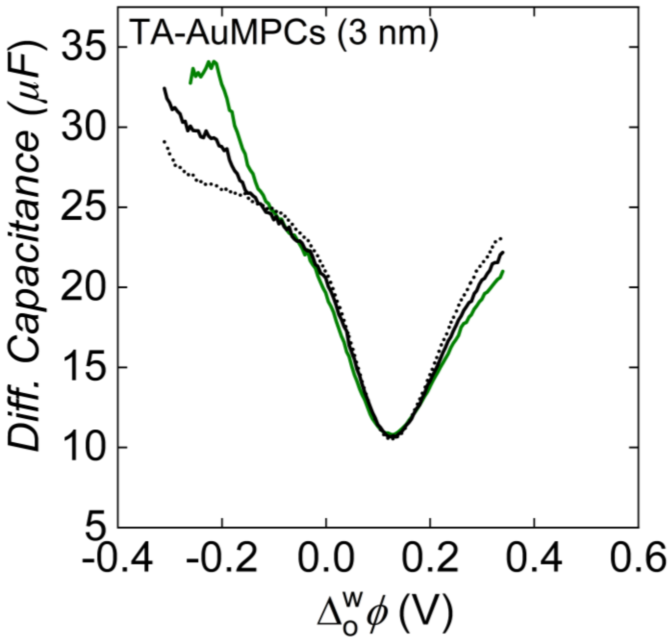

Figure S10. Differential capacitance curves of a polarisable aqueous-organic interface before ($\boldsymbol{x}, \boldsymbol{y}, \boldsymbol{z}$: .0 mM, black dotted curve) and after the adsorption of different concentrations of TA-AuNPs 3 nm nanoparticles. 1.6 nM (black curve) and 3.2 nM (green curve).

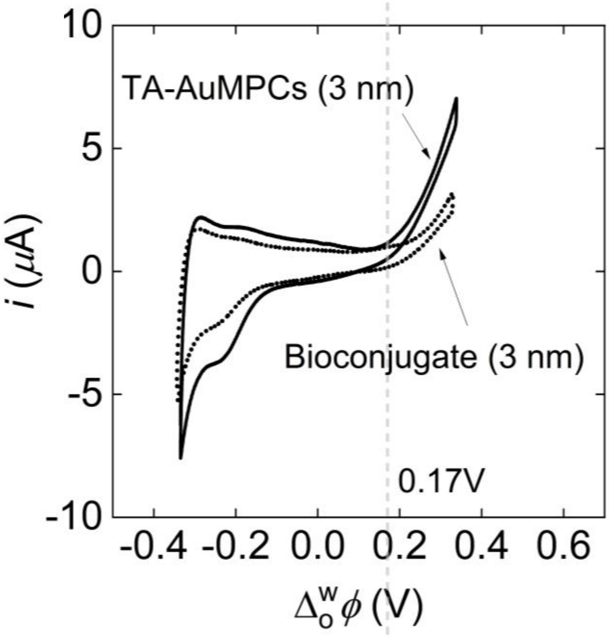

Figure S11. Cyclic voltammograms of 3 nm gold nanoparticles (black curve) and the bioconjugate (dotted curve) at an aqueous-organic interface and 1 mM DcMFc in the organic phase as electron donor. 20 mV s^{-1} scan rate. Electrochemical cell 2 configuration,

z: 1.6 nM TA-AuNPs and ***y:*** 20nM bioconjugates (A) CV performed with a narrow polarizable potential window.

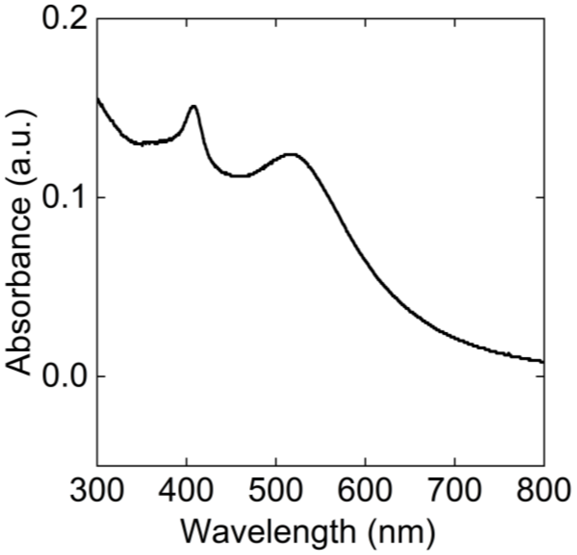

Figure S12. UV-Vis spectrum of 3 nm TA-AuNPs functionalized with Cyt c in 40 mM HEPES buffer, pH 7.5.

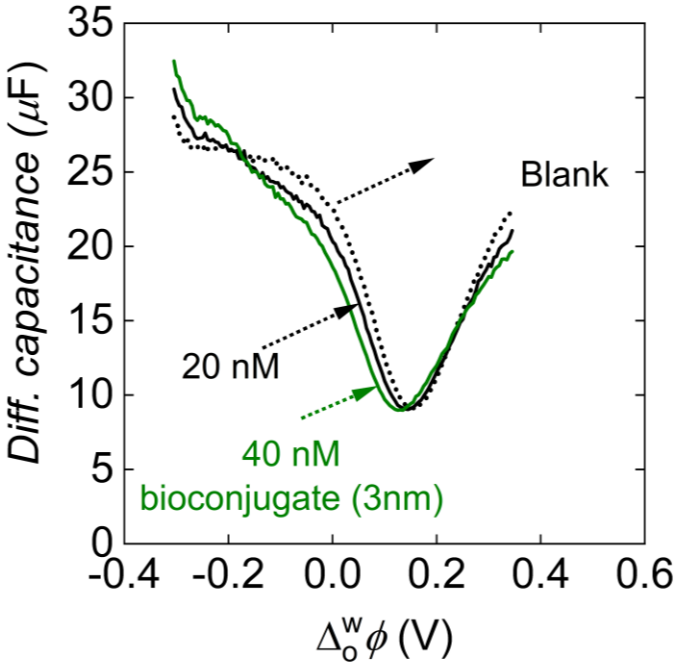

Figure S13. Differential capacitance curves of a polarisable aqueous-organic interface before ($\boldsymbol{x}, \boldsymbol{y}, \boldsymbol{z}$:.0 mM, black dotted curve) and after the adsorption of different concentrations of Cyt c-TA-AuNPs 3 nm bioconjugates, 20 nM (black curve), 40 nM (green curve).

References

1. Gamero-Quijano, A., Manzanares, J. A., Ghazvini, S. M. B. H., Low, P. J. & Scanlon, M. D. Potential-Modulated Ion Distributions in the Back-to-Back Electrical Double Layers at a Polarised Liquid|Liquid Interface Regulate the Kinetics of Interfacial Electron Transfer. *ChemElectroChem* **202201042**, (2022).

2. Brust, M., Walker, M., Bethell, D., Schiffrin, D. J. & Whyman, R. Synthesis of Thiol-derivatised Gold Nanoparticles in a Two-phase Liquid-Liquid System. *J. Chem. Soc., Chem. Commun.* 801–802 (1994) doi:DOI https://doi.org/10.1039/C39940000801.

3. Abad, J. M., Gass, M., Bleloch, A. & Schiffrin, D. J. Direct electron transfer to a metalloenzyme redox center coordinated to a monolayer-protected cluster. *J. Am. Chem. Soc.* **131**, 10229–10236 (2009).

4. Gamero-Quijano, A. *et al.* On the origin of chaotrope-modulated electrocatalytic activity of cytochrome c at electrified aqueous|organic interfaces. *Chem. Commun.* **58**, 3270–3273 (2022).

5. Haiss, Wolfgang; Thanh,Nguyen; Aveyar, Jenny; Fernig, D. Determination of Size and Concentration of Gold Nanoparticles from UV-Vis Spectra. *Anal. Chem.* **79**, 4215–4221 (2007).

6. Navarro, J. R. G. & Werts, M. H. V. Resonant light scattering spectroscopy of gold, silver and gold–silver alloy nanoparticles and optical detection in microfluidic channels†. *Analyst* **138**, 583–592 (2013).

7. Volkert, A. A., Subramaniam, V., Ivanov, M. R., Goodman, A. M. & Haes, A. J. Salt-mediated self-assembly of thioctic acid on gold nanoparticles. *ACS Nano* **5**, 4570–4580 (2011).
